# Viruses Infecting Reptiles

**DOI:** 10.3390/v3112087

**Published:** 2011-11-01

**Authors:** Rachel E. Marschang

**Affiliations:** Institut für Umwelt und Tierhygiene, University of Hohenheim, Garbenstr. 30, 70599 Stuttgart, Germany; E-Mail: rachel.marschang@googlemail.com; Tel.: +49-711-459-22468; Fax: +49-711-459-22431

**Keywords:** reptile, taxonomy, iridovirus, herpesvirus, adenovirus, paramyxovirus

## Abstract

A large number of viruses have been described in many different reptiles. These viruses include arboviruses that primarily infect mammals or birds as well as viruses that are specific for reptiles. Interest in arboviruses infecting reptiles has mainly focused on the role reptiles may play in the epidemiology of these viruses, especially over winter. Interest in reptile specific viruses has concentrated on both their importance for reptile medicine as well as virus taxonomy and evolution. The impact of many viral infections on reptile health is not known. Koch’s postulates have only been fulfilled for a limited number of reptilian viruses. As diagnostic testing becomes more sensitive, multiple infections with various viruses and other infectious agents are also being detected. In most cases the interactions between these different agents are not known. This review provides an update on viruses described in reptiles, the animal species in which they have been detected, and what is known about their taxonomic positions.

## 1. Introduction

Reptile virology is a relatively young field that has undergone rapid development over the past few decades. A number of factors have influenced the development of interest in these viruses. Many early studies dealt with reptiles as hosts for arboviruses that also infect humans as well as other mammals and birds, such as flaviruses and togaviruses. Interest in these viruses in reptiles has increased since the emergence of West Nile Virus in the western hemisphere at the end of the 20th century. A number of studies have shown that various arboviruses can infect a number of different reptile species and that temperature affects the development of viremia in these animals. Several studies have demonstrated that some arboviruses can persist in reptiles over winter, a factor that may play a role in the epidemiology of these viruses. Other studies have focused on the role of viruses as pathogens in reptiles. In many cases, however, Koch’s postulates have not been fulfilled for viral diseases of reptiles, so that the connection between virus and disease is postulated based on clinical, pathological and histological observations. Viruses that have been shown to be important pathogens in reptiles include ranaviruses and herpesviruses in chelonians, and paramyxoviruses in snakes. Other viruses, such as reoviruses, have not or have inconsistently induced disease in transmission studies, so that their role as primary pathogens has been questioned. However, a number of these viruses do appear to play a role in the health of reptiles, and may be part of factorial disease processes influenced by the viruses themselves, other infectious agents, and environmental factors. A third factor influencing interest in reptilian viruses is their taxonomic position and evolution. Analysis of the genomes of some reptilian viruses has placed a number of these in separate genera from viruses of other vertebrates, leading to proposals for new genera and, in some cases, a better understanding of coevolution of viruses and their hosts.

The detection and study of viruses of reptiles relies on a wide range of tools, including classical virological methods such as cell culture, as well as molecular methods, including PCR and sequencing as well as metagenomics. The combination of these methods has been successfully used to increase our understanding of viruses in this group of animals, although much remains to be learned, both about the viruses themselves and about their effect on the animals they infect.

The viruses that have been most commonly detected in reptiles include herpesviruses, especially in chelonians, adenoviruses, especially in lizards and snakes, reoviruses, especially in lizards and snakes, paramyxoviruses, especially in snakes, picornaviruses in tortoises, and iridoviruses, with ranaviruses detected predominantly in chelonians and invertebrate iridoviruses detected in lizards. Of these, the herpesviruses and adenoviruses have long been detected histologically based on the inclusion bodies they cause in tissues of infected animals, herpesviruses of tortoises, picornaviruses of tortoises, reoviruses, and iridoviruses can all be isolated in cell culture, and sensitive PCRs are available for the detection of herpesviruses, adenoviruses, paramyxoviruses, and iridoviruses.

This review aims to provide an overview over the viruses detected in reptiles to date as well as some information on the diseases associated with these viruses. The taxonomic positions of the reptilian viruses relative to viruses of other vertebrates are provided if information on this is available. The study of these viruses has grown beyond the point at which a full overview over every aspect of this topic could be provided in such a review, so that specific information on some aspects of reptile virology, particularly specifics on diagnostic testing and samples as well as serological aspects of infection, are only briefly mentioned for each virus family. The viruses in the review are presented according to taxonomic position, with DNA viruses (poxviruses, iridoviruses, herpesviruses, adenoviruses, papillomaviruses, parvoviruses, circoviruses, and a “tornovirus”) presented first, followed by RNA viruses (retroviruses, reoviruses, rhabdoviruses, paramyxoviruses, bunyaviruses, picornaviruses, caliciviruses, flaviviruses, togaviruses).

## 2. Poxviridae

Poxviruses are large double-stranded DNA viruses. Their morphology is somewhat pleomorphic, ranging from brick-shaped to ovoid with a lipoprotein surface membrane. Vertebrate poxviruses are currently grouped into eight different genera in the subfamily *Chordopoxvirinae*. These genera include viruses of mammals and birds [1]. Recently, a proposal has been made to form a new genus, named “Crocodylipoxvirus”, in the subfamily *Chordopoxvirinae* with *Nile crocodilepox virus* as the type species of the genus [2].

Poxviruses have been repeatedly detected in crocodilians with skin lesions around the world. The first report of poxvirus-associated disease in a reptile was in captive caimans (*Caiman crocodilus*) in the USA [3]. Similar cases have since been reported from caimans throughout the world [4,5]. Affected animals develop gray-white skin lesions on various parts of the body.

In Nile crocodiles (*Crocodylus niloticus*), poxvirus infections have been associated with brownish wart-like skin lesions that can occur over the entire body. Infection is associated with high morbidity but low mortality [6,7]. The genome of crocodilepox virus (CRV) has been completely sequenced [8]. It is 190,054 bp long and encodes a predicted 173 genes. The terminal regions are largely CRV specific, containing 48 genes that are unique among poxviruses. The central genomic region also contains multiple unique genes. Based on these observations and phylogenetic analysis of the genome sequence, Afonso *et al.* [8] suggested that CRV should be placed in a new genus in the subfamily *Chordopoxvirinae*. An atypical form of crocodile pox has also been observed in Nile crocodiles. This virus was associated with deeply penetrating skin lesions in farmed crocodiles in Africa. A PCR targeting a unique region of CRV, ORF 019, was developed and used to amplify a portion of the genome of the virus associated with these lesions. Analysis of the sequence of the PCR product (533 bp) showed that this virus was related to, but not identical with CRV [9].

Poxvirus infections have also been detected in individual cases in other reptiles by electron microscopy. Papular skin lesions around the eyes of a Hermann’s tortoise (*Testudo hermanni*) were found to contain pox-like viruses [10]. A flap-necked chameleon (*Chamaeleo dilepis*) in Tanzania was found to have two different types of intracytoplasmic inclusions in circulating monocytes. The inclusions were found to be caused by a Chlamydia-like organism and a pox-like virus [11]. A poxvirus infection in a tegu lizard was associated with brown papules on various parts of the body [12].

## 3. Iridoviridae

### 3.1. Virus Taxonomy

The iridoviruses belong to the Nucleo-Cytoplasmic Large DNA viruses (NCLDV), a group of DNA viruses that infect very diverse hosts. The NCLDV also include mimiviruses, phycodnaviruses, African swine fever virus, and poxviruses. Although the genome size of these viruses varies greatly (between 100 kb and 1.2 Mb), they appear to form a monophyletic group based on a subset of about 30 conserved genes [13]. Among these is the major capsid protein (MCP), which is conserved among several groups in the NCLDV. The MCP comprises 40% of the total virion protein in iridoviruses. Iridoviruses are 120 to 300 nm large with a double stranded DNA genome and an icosahedral capsid containing a lipid component. The family *Iridoviridae* is currently divided into five genera: *Iridovirus*, *Chloriridovirus*, *Ranavirus*, *Lymphocystivirus*, and *Megalocytivirus* [14]. Until recently, viruses of the genera *Iridovirus* and *Chloriridovirus* had only been described in invertebrates, while viruses of the genera *Ranavirus*, *Lymphocystivirus*, and *Megalocytivirus* are found in ectothermic vertebrates. Iridoviruses have been described as possible pathogens of reptiles since the 1960’s. The iridoviruses that have been described and partially characterized in reptiles include ranaviruses in chelonians, lizards and snakes; invertebrate iridoviruses (IIVs) in lizards; and erythrocytic necrosis viruses in lizards, snakes, and turtles.

### 3.2. Ranaviruses

Ranaviruses have been increasingly shown to be important pathogens of ectothermic animals. They have been regularly isolated from reptiles since the late 1990’s. They have been mostly described in chelonian species worldwide, including Russian tortoises (*Testudo horsfieldii*), eastern box turtles (*Terrapene c. carolina*) [15,16], Chinese softshell turtles (*Trionyx sinensis*) [17], Hermann’s tortoises (*Testudo hermanni*) [18], red-eared sliders (*Trachemys scripta elegans*), Burmese star tortoises (*Geochelone platynota*), gopher tortoises (*Gopherus polyphemus*), and Florida box turtles (*Terrapene carolina bauri*) [16], eastern box turtles (*Terrapene carolina carolina*) [19], Egyptian tortoises (*Testudo kleinmanni*) [20], a leopard tortoise (*Geochelone pardalis*) [21], marginated tortoises (*Testudo marginata*), and spur-thighed tortoises (*Testudo graeca*) [22]. In these species, viral infection has been associated with lethargy, anorexia, nasal discharge, conjunctivitis, severe subcutaneous cervical edema, ulcerative stomatitis, and “red-neck disease”. Histologically, infected animals have been found to have hepatitis, enteritis, and pneumonia. Basophilic intracytoplasmic inclusions have in some cases been described in epithelial cells of the gastrointestinal tract and hepatocytes of infected animals. In a transmission study with box turtles (*Terrapene ornata ornata*) and red-eared sliders, i.m. injection of a ranavirus isolated from a Burmese star tortoise led to disease, including lethargy, anorexia, ocular discharge, conjunctivitis, and oral plaques, and death of the animals in some cases [16]. Ranavirus infection has also been described in green pythons (*Chondropython viridis*) in Australia [23]. The snakes showed ulceration of the nasal mucosa, hepatic necrosis and severe necrotizing inflammation of the pharyngeal submucosa. In lizards, ranaviruses have been described in a gecko (*Uroplatus fimbriatus*) in Germany [24] and a mountain lizard (*Lacerta monticola*) in Portugal [25]. In the gecko, infection was associated with granulomatous lesions in the tail and liver. In the mountain lizard, no overt disease was documented. That lizard had a very high number of intracytoplasmic inclusion bodies in the erythrocytes indicative of infection with an erythrocytic necrosis virus, which was also detected by PCR.

The origin of infection in reptiles has not been documented. Characterization of the detected viruses has generally relied on sequencing of a portion of the highly conserved MCP gene. These analyses have shown the detected viruses to be closely related to frog virus 3 (FV3), the type species of the genus *Ranavirus*. The complete sequence of the soft-shelled turtle virus has been determined [26]. The genome is 105,890 bp in length. It has a high degree of sequence conservation and a collinear arrangement of genes with FV3. This analysis suggests that this reptilian ranavirus was transmitted from amphibians to reptiles. A study comparing genome sequences from a range of ranaviruses from amphibians and fish suggested that the ancestral ranavirus was a fish virus and that several recent host shifts have taken place, leading, among others, to infection of reptiles [27].

Spread of ranavirus infection within a mixed-species group of tortoises [22] as well as transmission from Burmese star tortoises to red-eared sliders [16] has shown that these reptilian ranaviruses can be transmitted between several different species. In a transmission study with Bohle iridovirus, this amphibian ranavirus was shown to be highly virulent in hatchling turtles (*Elseya latisternum* and *Emydura krefftii*) [28].

### 3.3. Invertebrate Iridoviruses

Until recently, viruses of the genus *Iridovirus* had only been described in invertebrates. These viruses are of particular interest because of their potential use for controlling important agricultural pests and vector insect species, in which they induce lethal infections [29]. At the end of the 1990’s two research groups in Germany isolated and characterized iridoviruses from crickets (Orthoptera, Gryllidae) of the species *Gryllus campestris* and *Acheta domesticus* [29] and *Gryllus bimaculatus* [30]. In both cases, the insects derived from commercial breeders that produced crickets as food for the pet trade. In one case, unusually high mortalities and extremely reduced fertility and life span were found in nymphs and adults of *Gryllus campestris* and *Acheta domesticus* from a Dutch breeder [29]. The infection was manifested by hypertrophy and bluish iridescence of the affected fat body cells. In infection studies on the host range cricket iridovirus (CrIV) was successfully transmitted to other Orthopteran species which included *Gryllus bimaculatus* [31].

CrIV was compared with IIV-6, the type species of the genus *Iridovirus*, on the basis of genome sequences and host range. No differences were detected between these two viruses in transmission studies with various insect species. Different gene loci including the MCP gene were analyzed, compared and led to the conclusion that CrIV and IIV-6 are not different species within the *Iridovirus* genus and that CrIV must be considered to be a variant and/or a novel strain of IIV-6 [31]. In the other case, an approximately 500 bp long region of the MCP gene of *Gryllus bimaculatus* iridovirus (GbIV) was sequenced. The obtained sequence was 97% identical to the corresponding sequence of IIV-6 [30].

IIVs belonging to the genus *Iridovirus* have only recently been described in reptilian hosts. In 2001 a German group reported the isolation of IIV-like viruses from the lung, liver, kidney, and intestine of two bearded dragons (*Pogona vitticeps*) and a chameleon (*Chamaeleo quadricornis*) and from the skin of a frilled lizard (*Chlamydosaurus kingii*) on viper heart cells (VH2) at 28 °C. The frilled lizard showed pox-like skin lesions and one of the bearded dragons had pneumonia. The other lizards had died with non-specific symptoms. A 500 bp portion of the MCP gene of the isolates was sequenced and had 97% identity to the nucleotide sequence of IIV-6 and 100% identity to the nucleotide sequence of GbIV. A host-switch of this virus from prey insects to the predator lizards was postulated [32].

An IIV was isolated from several tissues of a high-casqued chameleon (*Chamaeleo hoehnelii*). A 500 bp portion of the MCP gene was identical to the corresponding sequence of GbIV. The pathogenicity of this isolate for crickets of the species *Gryllus bimaculatus* was tested, resulting in mortality rates between 20 and 40%. Virus was reisolated from several fat body samples. In some fat bodies of infected crickets massive arrays of viruses could be detected by electron microscopy. These findings support the hypothesis that IIV from insects are able to infect reptiles [33]. IIV-like viruses have been detected in over 20 different lizards from various owners, as well as from crickets [34].

### 3.4. Erythrocytic Necrosis Viruses

Viral erythrocytic infections associated with irido-like viruses have been described in fish and amphibians as well as lizards, snakes, and turtles [35]. These viruses have been preliminarily classified as iridoviruses. They are associated with inclusions in erythrocytes of infected animals, and these inclusions were originally believed to be parasites (*Toddia* and *Pirhemocyton* sp.) [35,36]. Pathology associated with erythrocytic necrosis virus infections in reptiles is unclear, but morphological changes in infected erythrocytes have been documented. A transmission study conducted with the lizards *Lacerta monticola* and *Lacerta schreiberi* showed that infection with these agents can, in some cases, become systemic and may lead to death [37]. Recently, a PCR was successfully used to detect an iridovirus in a ribbon snake with erythrocytic inclusions in Florida, USA. The snake had hypochromic erythrocytes containing purple granular inclusions and pale orange or pink crystalloid inclusions and a necrotizing hepatitis. Transmission electron microscopy of the inclusions revealed particles morphologically consistent with members of the *Iridoviridae*. Approximately 60% of the erythrocytes examined in this snake were severely hypochromic and exhibited anisocytosis and polychromasia. Sequence analysis of a 628 bp portion of the DNA dependent DNA polymerase gene showed that this virus was distinct from other known iridoviral genera and species and that it may represent a novel genus and species in the family *Iridoviridae.* The virus was given the name *Thamnophis sauritus* erythrocytic virus (TsEV) [38]. A lizard erythrocytic virus (LEV) from a *Lacerta monticola* from Serra da Estrela, Portugal was also partially characterized based on a 596 bp length portion of the same gene. Analysis of the obtained sequence showed that it clustered together with the TsEV, with 65.2/69.4% nt/aa% homology with that sequence, although ultrastructural differences between the viruses were detected by electron microscopy [25]. This study supported the classification of the erythrocytic necrosis viruses of reptiles in a new genus in the family *Iridoviridae*. Their final classification and understanding of their relationship to erythrocytic necrosis viruses of amphibians and fish require further study.

## 4. Herpesviridae

### 4.1. Virus Taxonomy

Herpesviruses (HVs) are large, enveloped viruses with a double-stranded DNA genome and an icosahedral capsid. The family *Herpesviridae* is a member of the order *Herpesvirales*, which also contains the family *Alloherpesviridae*, which incorporates fish and frog viruses and the family *Malacoherpesviridae*, which currently contains a bivalve virus. The family *Herpesviridae* contains mammal, bird, and reptile viruses. It is divided into three subfamilies, the *Alpha*-, *Beta*-, and *Gammaherpesvirinae*. All of the reptilian HVs described so far belong to the family *Herpesviridae*, but none have yet been assigned to any of the existing genera [39]. It is considered probable that all vertebrates carry multiple HV species. In most cases, severe infection is only observed in very young or immunosuppressed animals or following infection of an alternative host. An important aspect of HV infections is their ability to establish life-long latent infections, which is assumed to be true of all HVs [40].

In reptiles, HVs have been detected in lizards [41–48], snakes [49,50], chelonians [51–63] and crocodylians [64,65]. None of the reptilian HVs has yet been assigned to a specific genus in the family *Herpesviridae*. A detailed analysis of available sequence information from two HVs from sea turtles, the green turtle HV (GTHV) and lung eye and trachea disease-associated HV (LETV) showed that these both originated from the lineage leading to the recognized members of the *Alphaherpesvirinae* and concluded that these viruses belong to a private clade in that subfamily. Analysis of the limited sequence data available from lizard HVs (an iguanid HV and three HVs of gerrhosaurs (plated lizards, GerHV1, GerHV2, and GerHV3)) did not yield a clear classification of these viruses, concluding that additional sequence data is necessary to clarify the relationships between these viruses and other HVs and to understand the evolution of these viruses [66]. Of the reptilian HVs, the chelonian HVs are most common and have been best characterized so far. The sequence information available is, however, generally limited to a small portion of the DNA polymerase gene. Analysis of this short fragment supports the classification of chelonian HVs in the subfamily *Alphaherpesvirinae* of the family *Herpesviridae* [57,61,62,66–69]. It has been suggested that the chelonian HVs form a monophyletic group typical of other genera in the family *Herpesviridae*, and that these viruses be classified together in a new genus with the suggested name “Chelonivirus” [57].

### 4.2. Herpesviruses in Squamates

A number of different HVs have been detected in lizards, while relatively few have been described in snakes. In snakes, HVs have been detected in venom glands of various species, in some cases associated with decreased venom production [50], and in a group of juvenile Boa constrictors that died with hepatic necrosis. In these cases, virus detection has been based on electron microscopy, and no data is available on the genomes of these viruses.

In lizards, a number of different HVs have been detected. HV-like particles, as well as particles resembling papova- and reoviruses were detected by electron microscopy in papillomas of green lizards (*Lacerta viridis*) [41]. Herpesviral DNA has also more recently been detected by PCR in tissues from papillomas from green lizards [48]. Based on the analysis of the partial DNA polymerase gene sequence obtained in that study, the authors speculated that the detected virus was related to HVs associated with fibropapillomatosis in sea turtles. However, the analytical support for that conclusion was not convincing, and the virus appeared to be more closely related to varanid HV 1, which is a probable member of the subfamily *Alphaherpesvirinae*, but does not cluster with the chelonid HVs [45].

A number of cases have been documented in which lizard HVs were associated with oral lesions in infected animals. The varanid HV 1 was detected in the oral mucosa and brain of green tree monitor lizards (*Varanus prasinus*) with proliferative stomatitis [45]. Three distinct HVs (gerrhosaurid HVs 1–3) were detected in Sudan plated lizards (*Gerrhosaurus major*) and a black-lined plated lizard (*Gerrhosaurus nigrolineatus*) with stomatitis. Analysis of a portion of the DNA polymerase gene of these viruses indicated that they may belong to the subfamily *Alphaherpesvirinae*, but that they appear to be only distantly related to other known HVs [44].

A few reports are available of HVs in lizards associated with lesions in the liver. A HV was detected by electron microscopy in hepatocytes of red-headed agamas (*Agama agama*) in the USA [42]. Iguanid HV 2 was detected in a San Esteban chuckwalla (*Sauromalus varius*). Partial sequence analysis of the DNA polymerase gene of this virus showed that it also appeared to belong to the *Alphaherpesvirinae*, but that an exact taxonomic classification was not possible based on the limited data available [43].

Iguanid HV 1 is the only lizard HV that has been isolated in cell culture. This virus was isolated from cell cultures derived from an infected green iguana (*Iguana iguana*). Transmission of the isolate to other lizards did not lead to the development of clinical signs [70]. In another case with a green iguana, HV-like particles were detected by electron microscopy in a lizard with hepatitis [46]. Neither of these viruses has been further characterized.

### 4.3. Herpesviruses in Crocodilians

HVs have been reported in crocodylians in two cases. The first was the detection of HV-like particles in the skin of saltwater crocodiles (*Crocodylius porosus*) in Australia with a crust on the abdominal skin [64]. However, a direct link between the lesions and the HV detected could not be drawn, as the animals with lesions were shown to also have a poxvirus infection, as well as bacterial infections precipitated by biting. In the USA, a HV was detected in American alligators (*Alligator mississippiensis*) with lesions in the cloaca [65]. Analysis of a short portion of the DNA polymerase gene from that virus indicated that it was very closely related to tortoise HV 1. However, since no histological changes typical of HV infection (e.g., inclusion bodies) were detected in the tissues, additional studies are necessary to prove infection and pathogenicity of this virus in crocodylians.

### 4.4. Herpesviruses in Chelonians

Of the reptilian HVs, the chelonian HVs are most common and have been best characterized so far. A number of reports are available on HV infection in water turtles. These have been described in Pacific pond turtles (*Clemmys marmorata*), painted turtles (*Chrysemys picta*), and map turtles (*Graptemys* spp.). Clinical signs reported in affected animals include lethargy, anorexia, and subcutaneous edema. Characteristic necropsy findings include hepatomegaly and pulmonary edema. Areas of hepatic necrosis with the presence of intranuclear inclusion bodies in hepatocytes were reported. Inclusions have also been demonstrated in the spleen, lungs, kidneys, and pancreas [58–60]. None of these viruses has been isolated and no further information is available on any of them.

Gray patch disease was one of the first HVs to be described in chelonians. It infects green sea turtles (*Chelonia mydas*). Aquaculture reared, 2- to 3-month-old turtles appear to be most commonly affected. The virus was described by electron microscopy, and no further data are available on the virus involved [51].

Lung, eye, and trachea disease (LETD) has also been described in green sea turtles. Clinical signs associated with infection are gasping, harsh respiratory sounds, buoyancy abnormalities, inability to dive properly, and the presence of caseated material on the eyes, around the glottis and within the trachea. Some of the infected turtles died after several weeks, while others became chronically ill. A HV (LETV) was isolated from diseased turtles in green sea turtle kidney cells and has since been further characterized [52,66,71].

In sea turtles, fibropapillomatosis has been associated with HV infection and has been described in many different species of marine turtles including green, loggerhead (*Caretta caretta*), Hawksbill (*Eretmochelys imbricata*), and olive ridley (*Lepidochelys olivacea*) sea turtles around the world. Infected turtles develop fibropapillomas and individual or multiple tumors can occur externally all over the body. Internal tumors are also possible. The viral etiology has been tested by tumor transmission using cell-free tumor extracts [72]. The fibropapillomatosis HV has never been isolated in cell culture. Analysis of a large portion of the genome of a fibropapilloma-associated turtle HV (FPTHV) showed that it is related to other alphaherpesviruses. Comparison of a number of different FPTHVs from different species, different locations and different years, showed that these were all closely related to one another, but that 4 variants (A, B, C, and D) could be differentiated. The viruses studied were most closely related to LETV [73]. In another study, sequencing of a large portion of the genome of a Hawaiian green sea turtle FPTHV and comparison of this virus to other FPTHVs from geographically and genetically diverse FP-affected marine turtles confirmed that these viruses should be classified as alphaherpesviruses, but placed in a genus separate from described HVs. The closest related virus was again found to be LETV. That study indicated that the FPTHVs can be divided into groups which cluster according to geographic origin, not host species [56].

Two HV-associated disease syndromes have been described in wild-caught loggerhead sea turtles (*Caretta caretta*); loggerhead genital-respiratory HV (LGRV), and loggerhead orocutaneous HV (LOCV). LGRV was associated with ulcers in the trachea, around the cloaca and on the base of the phallus, while LOCV was associated with ulcers in the oral cavity and cutaneous plaques which were covered with exudate and had an erythematous border as well as with pneumonia [57].

HV infections have been reported in many different species of tortoises (Testudinidae). Clinical signs commonly associated with infections include rhinitis, conjunctivitis, stomatitis and glossitis, which frequently develop into a diphtheroid-necrotizing stomatitis and glossitis, with diphtheroid membranes covering parts of the oral cavity and extending down into the trachea and esophagus. Edema of the neck is a common sign. Affected animals are generally anorexic and lethargic. Animals that survive acute HV infection may develop central nervous system disorders including paralysis or incoordination [74]. In a transmission study, spur-thighed tortoises inoculated with a tortoise HV either i.m. or intranasally developed disease signs consistent with HV infection [67].

Histologically, HV infections in tortoises may be associated with eosinophilic or amphophilic intranuclear inclusions in infected tissues, most frequently in epithelial cells of the tongue, oral mucosa, and upper respiratory tract as well as in the gastrointestinal tract. Occasionally, inclusions can also be found in epithelial cells of the urinary tract, in the brain, liver, and spleen. [74].

The ICTV lists a number of chelonid HVs as unassigned viruses in the family *Herpesviridae*. However, many of the viruses listed there were only described by electron microscopy, and no further characterization of the viruses is possible. Their relationship to one another and to newly described reptilian HVs can no longer be elucidated. A revised method of naming newly described and characterized chelonian HVs, particularly HVs of tortoises, has therefore been used in recent publications. The first tortoise HV from which sequence data from a small portion of the DNA polymerase gene became available, allowing a preliminary taxonomic analysis of the isolate, was from Russian and pancake tortoises (*Testudo horsfieldii* and *Malacochersus tornieri*) in Japan [69]. Studies on HVs from European tortoises showed that similar viruses can also be found in Russian tortoises in Europe, but that these viruses differ from the majority of isolates found in Mediterranean tortoises kept as pets in Europe, building two distinct genogroups [62]. Another distinct HV was detected in a California desert tortoise (*Gopherus agassizii*) in the USA [61]. A fourth tortoise HV has been detected in a bowsprit tortoise (*Chersina angulata*) in a zoo in the USA [63]. Naming of these various viruses has been confused due to the lack of a unifying method. Bicknese *et al.* [63] therefore proposed to name all of these HVs from tortoises “tortoise HVs” (THVs). In order to avoid confusion with other HVs and to conform to nomenclature of HVs in other animals, this name should be changed to “testudinid HVs” and the abbreviation should be changed to TeHV. The individual virus species were then numbered according to the date of publication, so that the Russian and pancake tortoise virus from Japan (and Europe) was named TeHV1, the California desert tortoise HV was named TeHV2, the HV most commonly found in Mediterranean tortoises in Europe was named TeHV3, and the bowsprit tortoise HV was named TeHV4. Some of these viruses have been shown to be able to infect multiple host species in the family Testudinidae. TeHV1 has mainly been described in Russian tortoises, but can also infect several other species. It is generally associated with relatively low morbidity and mortality rates [69,75]. TeHV3 has been detected in many different tortoise species, most commonly in spur-thighed tortoises (*T. graeca*), marginated tortoises (*T. marginata*), Hermann’s tortoises (*T. hermanni*), and Russian tortoises (*T. horsfieldii*). It is interesting that these species show different susceptibilities to HV-associated disease. TeHV3 infections in Hermann’s and Russian tortoises are generally associated with high morbiditiy and mortality, while spur-thighed tortoises appear to be relatively resistant to disease. This may be a reflection of the evolutionary history of the virus, and it has been speculated that this may be a virus of spur-thighed tortoises, and that infections of other tortoises represent host switches and are therefore associated with higher mortality rates. Antibodies against TeHV3 have also been detected in wild-caught spur-thighed tortoises in Turkey [76]. However, additional study is necessary to test this hypothesis.

## 5. Adenoviridae

### 5.1. Virus Taxonomy

Adenoviruses (AdVs) are the viruses most commonly identified in many species of lizards, particularly bearded dragons. The family *Adenoviridae* contains non-enveloped viruses with a genome consisting of linear, double-stranded DNA 26–45 kbp in size [77]. They have an icosahedral capsid made up mostly of non-vertex capsomers or hexons. The capsid also contains vertex capsomers (pentons) with fibers protruding from the virion surface. The fiber, hexon and penton are also the 3 major antigenic proteins found in AdVs [78]. AdVs occur worldwide and have been described from representatives of five classes of the Vertebrata [79]. Current taxonomy of the family *Adenoviridae* suggests a coevolutionary lineage of the viruses with their hosts, and additional host switches [78]. According to this theory, each genus of the virus family evolved within a different class of vertebrates. For mammals this would be the genus *Mastadenovirus*, for birds the genus *Aviadenovirus*, for reptiles the genus *Atadenovirus*, for amphibians the genus *Siadenovirus* and for fish the proposed genus “Ichtadenovirus”. However, recent findings on AdVs of chelonians and frogs shows that more work is necessary to fully understand the evolutionary relationships between these viruses and to fully appreciate their ability to switch hosts [80–82]. All AdVs described from squamatids so far are members of the genus *Atadenovirus* [83,84]. The genus *Atadenovirus* also contains viruses from ducks, geese, chickens, possums, and ruminants [78]. The complete genome of a single reptilian atadenovirus has been sequenced [85].

### 5.2. Adenoviruses in Reptiles

Although they are most commonly detected in various lizard species, particularly agamids, AdV infections have also been detected in many other reptile species including various species of snakes, chelonians, and crocodiles. AdVs appear to occur worldwide in captive populations and antibodies to AdVs have been detected in wild Boa constrictors from Costa Rica [86] and in wild-caught rattlesnakes from the USA [87]. A range of symptoms have been described in reptiles with AdV infection. The most common clinical sign in squamates is anorexia, which can also be associated with lethargy and wasting. Central nervous symptoms including head tilt, opisthotonus, and circling have been described [88–90]. In individual cases, stomatitis [91] and dermatitis [92] have also been described. The primary pathogenic role of AdVs has been questioned in many cases in which they were detected without signs of concurrent disease [93–96]. However, the pathogenicity of an AdV for reptiles was demonstrated in one case by an experimental transmission study [88]. In that study, a neonatal Boa constrictor was inoculated intracoelomically with an AdV isolated from a Boa constrictor with hepatic necrosis. The inoculated animal died 14 days p.i. with hepatic necrosis. Pathological changes described in AdV infected animals often involve only the liver, which may be enlarged with petechia or pale areas scattered throughout. The most common histological change described in infected animals is hepatic necrosis. The intestine is also frequently affected, and documented changes include dilation of the duodenum and hyperemia of the mucosa. Basophilic intranuclear inclusions are commonly described, particularly in hepatocytes where they have been associated with areas of necrosis. Intranuclear inclusions have also been documented in enterocytes [89,91,97], myocardial endothelial cells [93], renal epithelial cells [98], endocardium, and epithelial cells of the lung [96], as well as in glial and endothelial cells in the brain [90].

AdVs have only relatively recently been detected in several species of chelonians. In Sulawesi tortoises, infection was associated with severe systemic disease and a very high mortality rate (82%). Pathological findings in infected tortoises were multifocal hepatic necrosis, amphophilic to basophilic intranuclear inclusions and diffuse hepatic lipidosis, myeloid necrosis in bone marrow and severe necrotizing enterocolitis. The virus detected in these tortoises differed distinctly from the AdVs characterized from squamates so far and was determined to belong in the *Siadenovirus* genus [81]. The same virus (Sulawesi tortoise AdV-1) has also been found in exposed impressed tortoises (*Manouria impressa*) and a Burmese star tortoise (*Geochelone platynota*) [99]. A single case of AdV infection has also been reported in a leopard tortoise that was also infected with a herpesvirus. This animal had biliverdinuria, wasting, and episodes of haemorrhages [100]. In Hungary, an AdV was detected in a box turtle (*Terrapene ornata ornata*) with degeneration of liver cells, pronounced vacuolization of the cytoplasm, pyknosis of nuclei, and inclusion bodies in some hepatocytes. The virus detected in this animal differed distinctly from all previously described AdVs from reptiles [82].

A number of AdVs from reptiles have been isolated in cell culture, which has facilitated further characterization of individual viruses. However, many of the reptilian AdVs have not yet been successfully grown in cell culture. Snake AdVs have been isolated in several cases. Jacobson *et al.* [88] and Marschang *et al.* [86] each obtained AdVs from Boa constrictors (*Boa constrictor*) while Ahne and his co-workers isolated an AdV strain from a royal python (*Python regius*) [95] and from a moribund corn snake (*Pantherophis guttatus*) showing clinical signs of pneumonia [101]. This corn snake isolate (Snake AdV-1, SnAdV-1) was later randomly cloned and completely sequenced [85,102] and thus serves as a prototype for reptilian AdVs. Sequence evidence suggests that the SnAdV-1 and the isolate from a Boa constrictor [86] are identical. Recently, a different snake AdV was isolated from a cornsnake [103]. Although adenovirus infections are frequently described in lizards, there is only one report of the isolation of AdVs from helodermatid lizards in cell culture so far [104].

It has been hypothesized that atadenoviruses have co-evolved with reptiles and that atadenovirus infections in non-reptilian hosts represent host switches. This theory has also been supported by the finding that atadenoviruses of non-reptilian hosts have a biased genome with a relatively high AT content, which led to the naming of this genus. Another observation in many squamate AdVs is their relative species specificity: specific lizard AdVs are mostly found in a single host. However, there are a number of exceptions to this apparent rule: Eublepharid AdV-1 has been detected in two gecko genera in the subfamily Eublepharinae [83]. SnAdV-1 has been found in more than one superfamily of snakes in the infraorder Serpentes [86]. SnAdV-2 has been found in two genera of colubrid snakes and in viperid snakes [105,106]. Helodermatid AdVs appeared to be very species specific with Helodermatid AdV-1 found in Gila monsters (*Heloderma suspectum*) in both the USA and Europe, and the related but distinct Helodermatid AdV-2 found in beaded lizards (*Heloderma horridum*) [83,104]. However, a virus with 99% identity to Helodermatid AdV-1 (EU914207) in a partial sequence of the DNA polymerase gene has also been detected in liver tissue of a western bearded dragon (*Pogona minor minor*) in Australia [107].

The AdV most commonly described in bearded dragons, Agamid AdV-1, has been shown to consist of a number of slightly different viruses. In one study, which compared bearded dragon isolates based on partial DNA polymerase sequences slight differences were detected between different cases from different countries (USA / Germany / Austria / Hungary) [80]. A genotype differentiation of Agamid AdV-1 in bearded dragons in the USA based on a 253–256 bp portion of the hexon gene demonstrated four different genotypes. Concurrent infection with multiple genotypes was possible [108].

## 6. Papillomaviridae

Papillomaviruses are non-enveloped viruses 55 nm in diameter. The genome of the viruses is a single molecule of circular dsDNA. The papillomaviruses are highly host specific and tissue-restricted. They generally cause benign tumors (warts, papillomas) in their natural host. Occasionally, they can also cause these in lesions in related species. Sixteen different genera have been defined in the family *Papillomaviridae* so far, all with mammalian or avian hosts [109]. The first description of a papilloma-like virus (papovavirus) in reptiles was in wart-like skin lesions in a European green lizard (*Lacerta viridis*). The virus was identified by electron microscopy based on morphological characteristics. Herpes-like and reo-like viruses were also identified in the lesions [41]. Since then, papillomaviruses have been described in a number of cases in chelonians, including Bolivian side-neck turtles (*Platemys platycephala*) [110], a Russian tortoise (*Testudo horsfieldii*) [111], a loggerhead turtle (*Caretta caretta*) and a green turtle (*Chelonia mydas*) [112]. The Bolivian side-neck turtles had circular papular skin lesions that in some cases progressed to areas of necrosis and viral particles were detected by electron microscopy in skin biopsies [110]. The Russian tortoise had a history of stomatitis and papillomavirus-like particles were detected in a lung wash (but not in oral scrapings) by electron microscopy [111]. Lesions in the loggerhead turtle and green turtle associated with the papillomavirus infections were similar to those described in the Bolivian side-neck turtles and consisted of small white papules that resolved after several months. Analysis of partial sequence of the E1 protein gene revealed that these two viruses were distinct from one another and from previously described papillomaviruses. The viruses were named *Caretta caretta* papillomavirus 1 (CcPV-1) and *Chelonia mydas* papillomavirus 1 (CmPV-1) [112]. The complete sequence of both viruses has been determined. Analysis of the sequence shows that both chelonian papillomaviruses share a similar genome organization, and that this differs from that of other papillomaviruses in a number of aspects. Phylogenetic analysis based on concatenated amino acid and nucleotide sequences of 4 ORFs (E1, E2, L2, and L1) showed that the turtle papillomaviruses clustered together with avian papillomaviruses in a distinct clade separate from all mammalian papillomaviruses. The calculated papillomavirus tree was consistent with the idea that papillomaviruses have co-speciated with their host animals. However, nucleotide base substitution rates appeared to be slower for the chelonian papillomaviruses than those estimated for mammalian papillomaviruses. This was hypothesized to be due to slower metabolic rates and longer generation times in chelonians [113].

## 7. Parvoviridae

The parvoviruses are small (18–22 nm) non-enveloped round viruses with icosahedral symmetry and a single-stranded DNA genome. The family is divided into two subfamilies, *Parvovirinae* and *Densovirinae*. Viruses in the subfamily *Densovirinae* infect arthropods, while all vertebrate parvoviruses classified so far belong in the subfamily *Parvovirinae*. This subfamily has been suggested to be divided into at least seven different genera based on biological, genomic, phylogenetic, and serological features. These genera include the genus *Dependovirus*, which includes the only classified reptile parvovirus, serpentine adeno-associated virus, which is suggested to be a species within this genus [114]. Dependoviruses are generally associated with a helper virus (adeno- or herpesviruses).

In reptiles, parvo-like viruses were first described by electron microscopy in the duodenum of a four-lined rat snake (*Elaphe quatuorlineata*) and of an Aesculapian snake (*Elaphe longissima*), both with gastrointestinal disease. Adeno-like viruses were also detected in the duodenums of both snakes. Herpes- and picorna-like viruses were also detected in the duodenum of the Aesculapian snake [91]. Co-infections of adenovirus- and parvovirus-like viruses have been described repeatedly from both snakes and lizards with clinical signs including gastrointestinal disease as well as neurological signs and pneumonia [97,115]. In bearded dragons with neurological signs, parvo-like and adeno-like viruses were detected in enterocytes, as were *Isospora* sp. [89]. A parvovirus was isolated from tissues of a corn snake (*Pantherophis guttatus*) in iguana heart cells (IgH2), but was not further characterized [116]. Both adeno- and parvoviruses were isolated in cell culture from a Boa constrictor and a ball python (*Python regius*). Both parvoviruses were found to be identical and were given the name serpentine adeno-associated virus (SAAV). The genomic organization of this virus was determined to be most similar to that of described dependoviruses with two open reading frames, one encoding the putative non-structural (Rep1 and Rep2), and one encoding the capsid (VP1, VP2, and VP3) proteins. It is unclear whether SAAV can replicate without a helper virus, as both isolates were obtained together with adenoviruses [117]. SAAV has also been detected by PCR in an Indonesian pit viper (*Parias hageni*) infected with an adenovirus [106].

## 8. Circoviridae

Circoviruses are small, non-enveloped single-stranded DNA viruses with a circular genome. The family is currently divided into two genera, *Circovirus*, the type species of which is porcine circovirus, and *Gyrovirus*, the type species of which is chicken anemia virus. A single circo-like virus has been reported in macrophages of a painted turtle (*Chrysemys* sp.) with multifocal areas of necrosis in the spleen and liver. The virus was identified based on electron microscopy [118].

## 9. Tornovirus

A novel single-stranded DNA virus with a circular genome approximately 1800 nucleotides long was detected in two green sea turtles (*Chelonia mydas*) with fibropapillomatosis using metagenomics. This virus has a circular genome, with a hypervariable region and a conserved region. Numerous quasispecies were identified in both turtles. Most of the genome has no similarities to any known viruses. A single ORF (ORF2) has weak (25%) amino acid level similarity to the VP2 protein of chicken anemia virus. The virus was named sea turtle tornovirus 1 (STTV1). The authors of the study postulated that STTV1 might represent a new viral genus of the family *Circoviridae* or even a new viral family. Both of the infected turtles were severely afflicted with fibropapillomatosis with extensive external fibropapillomas and internal fibromas. STTV1 was detected in the fibropapillomas as well as in external swabs from the conjunctiva, oral cavity, cloaca, unaffected skin, and numerous internal tissues. The herpesvirus FPTHV was also detected in the fibropapillomas, but not in other tissues. STTV1 was also detected in leeches collected from one of the green sea turtles. STTV1 is not believed to be the cause of fibropapillomatosis, as it is not found in all fibropapillomatosis affected turtles, but both of the STTV1 positive turtles had severe fibropapillomatosis, and it was hypothesized that STTV1 might affect the immune system of infected sea turtles or that it might be an opportunistic pathogen [119].

## 10. Retroviruses

### 

*Retroviridae* virions are spherical, enveloped, and 80–100 nm in diameter. The genome of members of the subfamily *Orthoretrovirinae* consists of linear, positive sense single-stranded RNA which is reverse transcribed to cDNA early in the replication cycle. Historically, retrovirus nomenclature was based on electron microscopy and classified members of the genera *Alpharetrovirus* and *Gammaretrovirus* as C-type viruses (assembly of immature capsids at the plasma membrane) and members of the genus *Betaretrovirus* as A-type particles (immature capsids) in the cytoplasm. A-type particles then budded with either B- or D-type morphology [120]. Retroviruses are widely distributed among vertebrates as exogenous infectious agents. Endogenous proviruses resulting from infection of germline cells also occur widely among vertebrates. Retroviruses have been repeatedly found in reptiles. Retroviral particles with C-type morphology were originally described from a Russell’s viper (*Vipera russelli*) with a sarcoma [121], named viper retrovirus (VRV) and from a corn snake with a rhabdomyosarcoma (corn snake retrovirus, CSRV) [122]. VRV has been classified as a *Gammaretrovirus*, the type species of which is murine leukemia virus [120]. Two morphologically distinct retrovirus-like particles were detected in the venom glands of a number of *Bothrops jararacussu* that were apparently healthy [123]. C-type particles have also been described in Burmese pythons (*Python molurus bivittatus*) with various neoplasms [124]. A type-A-like retrovirus was detected in metastatic intestinal epithelial cells in the liver of an emerald tree boa (*Corallus caninus*) with adenocarcinoma [125]. Retroviral sequences have been detected in the genomes of many different reptiles. Systematic searches for sequences of murine leukemia related retroviruses have shown that related viruses can be found in a wide range of animals including reptiles, amphibians, birds, and mammals. In reptiles these viral sequences have been detected in the genomes of a number of chelonians, squamates, and a sphenodon (tuatara) [126,127]. A different, highly divergent endogenous retrovirus has also been described in a tuatara. The virus could not be placed in any known retroviral genus, but phylogenetic analysis placed it at the base of the spumavirus clade. This virus has been named Sphenodon endogenous virus (SpeV) [128]. A study on endogenous retroviral sequences from crocodilians showed that the genomes of several different species from different families in the order Crocodylia contained retrovirus sequences that were related to one another but highly divergent from other members of the *Retroviridae* [129]. Further study on the distribution and phylogenetic relationships of crocodilian endogenous retroviruses (CERVs) detected CERVs in 20 extant crocodilian species. The CERVs detected clustered into two major clades (CERV1 and CERV2). CERV1 was found only in crocodiles (family Crocodylidae) and clustered as a sister group of the genus *Gammaretrovirus*, while CERV2 clustered distantly to all known retroviruses [130]. Retroviruses have been repeatedly isolated from boid snakes with inclusion body disease (IBD) [131,132]. No sequence information is available from these viruses so that an exact classification of the isolated viruses has not been carried out. A retrovirus was isolated from a Burmese python (*Python molurus*) that was kept together with IBD positive pythons. The viral genome was fully sequenced and found to be distantly related to both B and D types and the mammalian C type viruses, but the virus could not be classified. Further study indicated that this is a highly expressed endogenous virus of Burmese pythons that is not associated with IBD. A similar virus was also identified in blood pythons (*Python curtus*). These viruses were named python endogenous retrovirus (PyERV) [133].

#### Inclusion Body Disease (IBD)

Inclusion Body Disease (IBD) is a disease of snakes that has been described worldwide in captive snakes. The disease is characterized by the formation of intracytoplasmatic inclusions in neurons and in epithelial cells of various organs. The inclusions are made up of protein. IBD generally affects boid snakes, although it has also been reported in palm vipers (*Bothriechis marchi*) and an eastern king snake (*Lampropeltis getulus*) [118,134]. Clinical signs associated with IBD are very variable. They include regurgitation followed by anorexia, and neurological symptoms including incoordination, disorientation, head tremors, lethargy and death. Pythons primarily show signs of CNS disease, while boas may show signs of CNS disease and/or regurgitation. Other clinical signs that have been associated with IBD include pneumonia, undifferentiated cutaneous sarcomas, lymphoproliferative disorders, and leukemia. Animals with inclusions in various tissues but without apparent signs of disease have been described, particularly boas. The disease is believed to be of viral etiology, and retroviruses have been implicated as a possible factor [131,132]. However, Boa constrictors have also been shown to harbor endogenous retroviruses [127], making a definitive connection between retroviruses found in IBD positive snakes and disease difficult. There have been several studies on the transmission of IBD. In one study, two snakes injected with a retrovirus isolate from an IBD positive snake developed clinical symptoms within 8 days of inoculation. However, no virus was reisolated from the diseased snakes [132]. In another study, four non-infected boas received inoculations of liver homogenate from a snake with IBD; inclusions (but not disease) developed 10 weeks later [135]. The inclusions observed in tissues of IBD positive snakes have been shown to consist of a unique 68-kd protein [135]. The origin of the protein remains unknown.

## 11. Reoviridae

### 11.1. Virus Taxonomy

The family *Reoviridae* consists of non-enveloped viruses 60 to 80 nm in diameter with a segmented double-stranded RNA genome. The family contains 12 genera including the genera *Orthoreovirus*, *Rotavirus*, *Orbivirus*, *Coltivirus*, *Seadornavirus*, and *Aquareovirus*, which contain vertebrate viruses. All of the reoviruses detected in reptiles have been tentatively classified in the genus *Orthoreovirus*. These viruses infect only vertebrates and are spread by the respiratory or fecal-oral route. All members of the genus have ten RNA segments which can be divided into large (L), medium (M), and small (S) size-classes [136]. While the most common human orthoreovirus, Mammalian Reovirus, is not typically associated with significant disease, the majority of *Orthoreovirus* species have been shown to cause significant and often fatal disease in birds, and primates. Five species have been classified in the genus *Orthoreovirus*: Mammalian orthoreovirus, Avian orthoreovirus, Nelson Bay orthoreovirus, Baboon orthoreovirus, and Reptilian orthoreovirus. All but the mammalian orthoreovirus isolates are fusogenic in cell culture [136]. Sequences obtained from the S1 and S3 genome segments of a python reovirus showed that this virus represents a separate species in the genus *Orthoreovirus* [137]. Comparison of a 75 amino acid sequence of the RNA-dependent RNA polymerase of reoviruses isolated from different snake species, lizard species, and a tortoise supported the classification of all of the studied viruses in the genus *Orthoreovirus*. The reptilian viruses were monophyletic and formed three distinct clusters [138,139]. A reovirus detected in the liver of a rough green snake (*Opheodrys aestivus*) was found to be a novel strain distinct from the previously described reptilian orthoreoviruses at a level consistent with species difference based on the same partial sequence of the RNA-dependent RNA polymerase [140].

### 11.2. Reoviruses in Reptiles

Reovirus infections have been relatively frequently described in reptiles. In lizards, reoviruses have been described by electron microscopy associated with papillomas in green lizards (*Lacerta viridis*) [41]. Isolation of reoviruses from lizards has been described from green iguanas (*Iguana iguana*) that died with no specific clinical signs in Germany [141] and from a spiny-tailed lizard (*Uromastyx hardwickii*) in the United Kingdom that died during a disease outbreak associated with changes in the lungs [142]. An outbreak of reovirus-associated enteropathy and hepatopathy was described in leopard geckos (*Eublepharis macularius*) in the USA [143]. Serological tests have provided evidence of reovirus infections among other lizards as well, including wild lizards in Central America [144,145].

In snakes, reoviruses have been isolated from Chinese vipers (*Azemiops feyi*) with enteritis, from a ball python (*Python regius*) [146], an emerald tree boa (*Corallus caninus*) [147], an Aesculapian snake (*Elaphe longissima*) [141], from the brain of a prairie rattlesnake (*Crotalus viridis*) with signs of central nervous system (CNS) disease [148], and from rough green snakes (*Opheodrys aestivus*) in the USA with a necrotizing hepatitis [140]. Rough green snakes imported into Hungary from the USA were also found to be infected with an orthoreovirus [149]. Reoviruses were isolated from Moellendorff’s ratsnakes (*Elaphe moellendorffi*) and beauty snakes (*Orthriophis taeniurus*) with fatal respiratory disease. The virus isolated from that outbreak was inoculated intratracheally, orally and nasally into a black ratsnake (*Elaphe obsoleta obsoleta*). That snake died with pneumonia 26 days p.i. A reovirus was reisolated from the lungs of the dead snake [150]. Reoviruses were isolated from three Boa constrictors with inclusion body disease (IBD) in Germany [151]. A transmission study was carried out using one of these isolates. Juvenile Boa constrictors were inoculated intratracheally, intraperitoneal, and per os with virus suspension. No specific disease or pathology was observed in the infected animals, although virus was reisolated from the infected snakes up to 18 weeks p.i. [152].

In tortoises, a reovirus has only been isolated in one case from a spur-thighed tortoise (*Testudo graeca*). The tortoise was cachectic and had a necrosis of the epithelium of the tongue [153].

## 12. Rhabdoviridae

*Rhabdoviridae* is one of the families in the order *Mononegavirales*. The rhabdoviruses are enveloped, 100–430 nm long and 45–100 nm in diameter. Viruses infecting vertebrates are bullet or cone-shaped. The genome consists of a single strand of negative sense RNA. Some of the rhabdoviruses are arboviruses, while others multiply only in mammals, or birds, or fish, or invertebrates, or plants and certain plant-feeding arthropods. Some of the vertebrate viruses have a very broad host range. The family *Rhabdoviridae* is currently divided into six genera [154].

In reptiles, rhabdovirus infections have been documented based on both serology and virus isolation. Little is known about the importance of rhabdoviruses in reptiles, but the rhabdoviruses detected in reptiles so far have been arboviruses that were also capable of infecting mammals. Antibodies against a vesicular stomatitis virus have been detected in wild-caught reptiles in Texas, USA [155]. Neutralizing antibodies against Bahia Grande Virus, a rhabdovirus isolated from mosquitoes in Texas were detected in a single reptile (from 100 tested) from Texas. Antibodies against this virus were also detected in humans, cattle, and sheep [156]. Charleville virus, an unassigned rhabdovirus, has been isolated from heart, liver and lung of an Australian house gecko (*Gehyra australis*) in Australia. The virus was isolated in mice. Antibodies to this virus were also detected in a human [157]. Marco, Timbo, and Chaco viruses have been isolated from the teiid lizard *Ameiva ameiva ameiva* in Brazil. Chaco virus was also isolated from another species of teiid lizard, *Kentropyx calcaratus* from the same area. All three viruses were isolated in suckling mice. Both Marco and Chaco viruses were also shown to multiply in the salivary glands of the mosquito *Aedes aegypti* and could be passaged serially in these mosquitoes [158]. Marco, Timbo, and Chaco viruses were found to replicate to highest titer at 30 °C and were identified as rhabdoviruses based on morphology by electron microscopy [159]. A further uncharacterized rhabdovirus was detected by electron microscopy in intracytoplasmic inclusions in erythrocytes of another South American teiid lizard, the caiman lizard (*Dracaena guianesis*) [160].

## 13. Paramyxoviridae

### 13.1. Virus Taxonomy

The family *Paramyxoviridae* contains viruses with a negative sense single-stranded RNA genome. Virions are 150 nm or more in diameter, pleomorphic, and consist of a lipid envelope surrounding the nucleocapsid. The family is currently divided into the subfamily *Paramyxovirinae* with the genera *Rubulavirus*, *Avulavirus*, *Respirovirus*, *Henipavirus* and *Morbillivirus* and the subfamily *Pneumovirinae* with the genera *Pneumovirus* and *Metapneumovirus*. Members of the subfamily *Paramyxovirinae* encode 7 to 9 proteins. Virion proteins common to all genera include three nucleocapsid-associated proteins: an RNA-binding protein (N), a phosphoprotein (P), and a large polymerase protein (L); three membrane associated proteins: a matrix protein (M), a fusion protein (F) and an attachment protein (G, H, or HN). These genes are in the order 3′ N-P-M-F-HN-L 5′ in the genomes of members of this subfamily. There is, however, diversity in genome length, presence or absence of overlapping open reading frames (ORFs) within these common genes, genomic terminal sequences, and length and sequence of intergenic and untranslated regions. Among the genes common to all members of the *Paramyxovirinae*, the sequence relatedness between corresponding proteins is greater for N, P, M, and L than for F and HN, which are less conserved. Some, but not all members of this family have hemagglutinating and neuraminidase activity [161].

Paramyxoviruses (PMVs) have been isolated in cell culture from a number of different reptiles. The first PMV isolated from a snake, Fer-de-Lance virus (FDLV) is considered the type species of the reptilian PMVs. All related reptilian PMVs have hemagglutination and neuraminidase activity. The complete genome of FDLV has been sequenced and analyzed. It has a length of 15,378 nt. The genome encodes seven discrete, non-overlapping transcription units, one of which contains two overlapping ORFs. The genome organization is: 3′ N-U-P/V-M-F-HN-L 5′, with N, M, F, HN, and L defined as above. U encodes a protein of unknown function with no similarity to known sequences and P/V contains ORFs for paramyxoviral P and V genes. The analysis of the complete sequence showed that this virus should be classified in the subfamily *Paramyxovirinae* within a new genus with the proposed name *Ferlavirus* [162]. It has been proposed to expand the *Paramyxovirinae* subfamily to include the genus *Ferlavirus*, with FDLV as the type species [163].

### 13.2. Paramyxoviruses in Reptiles

PMV are an important cause of disease in snakes in both private and zoologic collections and have been described in many different parts of the world [164–167]. The first PMV outbreak in snakes was described in 1976 in a serpentarium in Switzerland [164]. The virus isolated during that outbreak was FDLV, which is the type species of the genus *Ferlavirus* [168]. In snakes, PMV infections are most commonly associated with respiratory disease, but CNS disease is also regularly observed. Frequent clinical signs are respiratory distress, head tremors, regurgitation, anorexia, and sudden death. Severe disease is most commonly observed in viperid snakes, often as outbreaks, but PMV have been found in snakes from the families Boidae, Elapidae, Colubridae, and Crotalidae [118]. In one case, Koch’s postulates have been fulfilled for pulmonary lesions associated with PMV infection in Aruba Island rattlesnakes (*Crotalus unicolor*). In that study, snakes were inoculated intratracheally with a PMV cell culture isolate from an Aruba Island rattlesnake. Several snakes developed pulmonary symptoms including blood in the lungs, trachea and oral cavity. The two animals that were not euthanized earlier died 19 and 22 days p.i. [169]. Lungs of diseased snakes are generally congested and hemorrhagic. Histology often shows proliferative interstitial pulmonary disease with proliferation and vacuolation of epithelial cells lining the faveoli. In some cases, intracytoplasmic inclusions can be seen in lining epithelial cells [165,169].

In lizards, PMV infections have been described much less frequently than in snakes. However, antibodies against PMV have been detected in both wild-caught and captive lizards [144,145,170]. Isolation of PMV from lizards is relatively rare [144,145,171] and only once have clinical symptoms of pneumonia been described in PMV infected caiman lizards (*Draecena guianensis*). These animals showed few clinical signs and were either found dead or were anorexic prior to death. Pathological and histological examination showed proliferative interstitial pneumonia [172].

Descriptions of PMV infections in chelonians are also rare. These infections have been associated with dermatitis [173], and pneumonia [174,175]. In a single case, a PMV was isolated from a Hermann’s tortoise (*Testudo hermanni*) with pneumonia. That virus was further characterized and found to be related to but distinct from the described ferlaviruses from snakes and lizards so far [174]. In another case, two distinct squamatid ferlaviruses were detected in a leopard tortoise (*Geochelone pardalis*) with pneumonia. The detected viruses were each most closely related to ferlaviruses from snakes. However, it was unclear whether the virus was a causative agent, as no virus was detected in the lungs of the diseased animal [175].

Comparisons of ferlaviruses have been carried out based on sequencing of various portions of the genome and on serological cross-reactivity. Hemagglutination inhibition (HI) assays were one of the first tools used to study the relationships among ferlaviruses and between ferlaviruses and other PMVs. In a study using antibodies raised in rabbits against three different snake PMV isolates (later identified to be members of subgroups A and B) and other PMVs to investigate antigenic relations, no cross reactivity was documented between the ferlaviruses and non-reptilian PMVs, but the three ferlaviruses did cross react [176]. Another study that investigated cross reactivity of several snake PMV isolates with avian PMV types demonstrated antigenic cross-reactivity between the snake isolates and avian PMV types 1, 3, and 7, in fewer cases, also to types 4 and 9 [177]. A PMV that serologically cross-reacted with avian PMV type 7, was also isolated from a reticulated python (*Python reticularis*) in the United Kingdom [178]. Studies based on sequencing of parts of the genome of a number of ferlaviruses have, however, all supported classification of these viruses separate from avian PMVs. Comparisons of 16 reptilian PMVs based on sequencing of 518 bp of the L gene and 352 bp of the HN gene have shown all studied viruses to cluster together with FDLV [179]. Further comparisons based on partial L gene (566 bp) and F gene (918 bp) sequences [180], 920 bp of the fusion protein gene [181], and a 679 bp region of the HN gene and a 627 bp region of the L gene [182] have all shown the described viruses to cluster with the ferlaviruses. Analysis of PMVs from snakes and lizards based on partial L, HN, and U gene sequences showed that the squamatid isolates all clustered into two closely related groups (“A” and “B”), with no indication of species specificity. A tortoise isolate was distinct from but related to these, based on partial L gene sequence, but the presence of a U gene could not be proven in this virus [174]. A third genogroup (“C”) within the genus *Ferlavirus* has also been described in squamatids [103].

## 14. Bunyaviridae

Bunyaviruses are spherical or pleomorphic enveloped viruses 80–120 nm in diameter. The viral genome comprises 3 molecules of negative or ambisense single-stranded RNA, which are called L (large), M (medium), and S (small). The family is currently divided into five genera: *Orthobunyavirus*, *Hantavirus, Nairovirus*, *Phlebovirus*, and *Tospovirus*. The first four of these are capable of infecting vertebrates. Orthobunyaviruses, nairoviruses, and phleboviruses replicate alternately in invertebrates. They are generally cytolytic for their vertebrate hosts, but cause little or no cytopathogenicity in their invertebrate hosts. Different viruses have different arthropod vectors including mosquitoes, ticks, phlebotomine flies and others [183].

A bunyavirus was isolated from the blood of a Texas soft-shelled turtle (*Trionyx spinifer emoryi*) which was wild-caught in Texas, USA. The virus was isolated in suckling mice by intracerebral inoculation and caused 100% mortality in inoculated mice. The virus was not further characterized, but was found to serologically cross-react with Cache Valley and Tensaw viruses, both mosquito-borne viruses of the genus *Orthobunyavirus* [154]. Kowanyama virus, an unassigned member of the family *Bunyaviridae*, has been isolated from both mosquitoes and a skink (*Ablepharus boutonii virgatus*) in Australia [184].

## 15. Picornaviridae

The family *Picornaviridae* belongs to the order *Picornavirales*, a group of viruses with a positive-sense RNA genome which is translated into an autolytically processed polyprotein, a pseudo T = 3 symmetry, and a three domain module [185]. The family *Picornaviridae* is currently divided into 12 recognized genera [186]. None of the reptilian picornaviruses has been taxonomically classified to date. The first report of picorna-like viruses in reptiles was based on the detection of *ca.* 20–27 nm large viral particles in a lattice or row formation in duodenal and splenic cells in a Boa constrictor and in duodenal cells in an Aesculapian snake (*Elaphe longissima*). Both snakes showed signs of gastrointestinal disease. An adeno-like virus was also found in the duodenum and spleen of the Boa constrictor, while parvo-, adeno-, and herpes-like viruses were detected in the duodenum of the Aesculapian snake [91]. Picorna-like viruses have been detected repeatedly by isolation in cell culture in a number of different tortoise species. These viruses have been grouped together based mainly on growth characteristics in Terrapene heart cells (TH-1; ATCC CCL-50), in which they cause lytic infection and are resistant to chloroform [187]. These viruses have been called Virus “X”. They have most frequently been isolated from spur-thighed tortoises (*Testudo graeca*), but are also found in marginated tortoises (*T. marginata*), Hermann’s tortoises (*T. hermanni*), leopard tortoises (*Geochelone pardalis*), and Egyptian tortoises (*T. kleinmanni*). They have been associated with various signs of disease in infected animals, including softening of the carapax in young animals, diphtheroid-necrotizing stomatitis, rhinitis, conjunctivitis, and ascites, but have also been isolated from clinically healthy animals [153,188]. In numerous cases, tortoises infected with Virus “X” have also been shown to be infected with other pathogens, especially herpesviruses and *Mycoplasma* spp. [189]. Heuser *et al.* [190] have now sequenced 7077 nt of the genome of a Virus “X” isolate from a spur-thighed tortoise. Analysis of the sequence indicates that this virus belongs to the family *Picornaviridae*, but differs significantly from described picornaviruses. It has therefore been proposed that this virus belongs to a novel picornavirus genus [191]. Sequencing of a large portion of the genome of one isolate will soon allow comparison of a number of isolates from different tortoise species displaying various clinical signs, allowing a better understanding of the phylogeny of these viruses, as well as their pathogenicity and epidemiology. It will also allow the development of better diagnostic assays in the future, as diagnosis currently relies exclusively on isolation in cell culture.

## 16. Caliciviridae

Caliciviruses (CVs) are non-enveloped, 27–40 nm in diameter and have icosahedral symmetry. The genome consists of a positive sense single-stranded RNA. The family is divided into four different genera. CVs infect a broad range of animals, and most individual CV species exhibit a natural host restriction. However, the vesicular exanthema of swine virus (VESV) in the genus *Vesivirus* is an exception, with a broad host range. Transmission is generally via contaminated food, water, fomites, and sometimes via aerosolization [192].

In reptiles, CVs have been isolated from four different snake species (Aruba island rattlesnakes (*Crotalus unicolor*), a rock rattlesnake (*C. lepidus*), and an eyelash viper (*Bothrops schlegeli*)), as well as from Bell’s horned frogs (*Ceratophrys orate*) in a single collection in the United States [193]. Some, but not all of the animals from which virus was isolated had enteritis and hepatitis. A transmission study with rattlesnakes (*Crotalus viridis helleri*) showed that these animals could be infected and virus could be reisolated (in one out of two snakes inoculated), but no pathognomonic disease was observed in the infected snake. Transmission of the virus to pigs led to seroconversion, but not disease. The isolated viruses were considered identical based on serological comparisons, but were serologically distinguishable from other CV serotypes. The isolated virus was therefore named reptilian calicivirus *Crotalus* type 1 (RCV Cro-1) [193]. Sequence analysis of a portion of the polymerase region of the genomes of this and other CVs showed that the reptilian CV is closely related to the San Miguel sea lion CVs [194]. Reptile calicivirus is now classified as a strain of vesicular exanthema of swine virus in the genus *Vesivirus* [192]. A PCR for the detection of RCV, as well as other vesiviruses has been described [195], but no information is available on the prevalence of this virus in reptiles.

## 17. Flaviviridae

### 

Flaviviruses are enveloped, single-stranded RNA viruses. The family *Flaviviridae* is divided into the genera *Flavivirus*, *Pestivirus*, and *Hepacivirus*. All of the flaviviruses described in reptiles belong to the genus *Flavivirus*, which contains viruses that are transmitted by hematophagous arthropods (arboviruses). Some flaviviruses are able to infect and replicate in a wide variety of vertebrate hosts (including mammals, birds, and reptiles) [196], and the focus of much interest in flaviviruses of reptiles has been the role of these animals as reservoirs for human disease. The emergence of West Nile virus (WNV) in the Western hemisphere in 1999 and the finding that this virus could infect a number of different reptile species increased interest and research in flaviviruses in reptiles.

Antibodies against various flaviviruses, including St. Louis encephalitis virus, Powassan virus, Japanese encephalitis virus, and WNV have been found in chelonians, snakes and crocodilians in various parts of the world [197–203]. Japanese encephalitis virus has been isolated from Chinese rat snakes in Korea [204]. Transmission studies with Japanese encephalitis virus have shown that lizards can be infected with this virus both by parenteral inoculation and, in some species, by feeding on infected mosquitoes. Infected animals develop viremia, and the development of viremia is temperature dependent [205]. No clinical symptoms were reported in the lizards in that study. Direct virus detection in naturally infected reptiles by isolation in cell culture or by RT-PCR has been described less frequently. In one report, a flavivirus-like virus was isolated from a leopard tortoise (*Geochelone pardalis*) with epistaxis, cloacal hemorrhage, biliverdinuria, and anemia. Herpes and adenoviruses were isolated from other animals in the same collection, and intraerythrocytic inclusions of unknown etiology were also described in these tortoises [100,206]. The most commonly described flavivirus in reptiles is WNV.

#### West Nile Virus (WNV)

WNV is a flavivirus that primarily cycles between mosquitoes and birds. It was first documented in North America in 1999 [207]. A number of transmission studies have been carried out with WNV and various reptile species. In a transmission study with green iguanas (*Iguana iguana*), Florida garter snakes (*Thamnophis sirtalis sirtalis*), red-eared sliders (*Trachemys scripta elegans*), and North American bullfrogs (*Rana catesbeiana*), the iguanas, snakes, and frogs became infected and the iguanas and frogs both developed low level viremias. No disease was reported in any of the infected animals [208]. In a transmission study with common garter snakes (*Thamnophis siralis*) that were infected subcutaneously, 5 of 9 infected snakes became viremic, while five had persistently low levels of neutralizing antibodies. Four of the experimental animals died and high titers of virus were found in multiple organ samples [209]. Another transmission study was able to induce a moderate viremia in four western fence lizards following inoculation with WNV [210].

WNV has been shown to be pathogenic for crocodilians and causes high titer viremias in these animals. Antibodies against WNV have been detected in crocodilians in many different countries, including in crocodiles at a commercial farm in Israel [200], in wild alligators in Florida [201], and in farmed crocodiles in Mexico [203]. In the USA, WNV has been detected in disease outbreaks in American alligators (*Alligator mississippiensis*) in a number of different states including Georgia [211], Florida [201], and Louisiana [212]. In Florida, the highest mortality was found in young animals. Neurological clinical signs included anorexia, tremors, swimming on their sides, spinning in the water, and opisthotonus. Oral lesions (stomatitis) were also noted. Death occurred 24–48 hours after the appearance of clinical signs. The highest viral loads were detected in the livers of affected animals [201]. In an outbreak among alligators in Georgia, neurological signs, including “star gazing” were also the predominant signs associated with infection, and oral lesions were also seen. Again, hatchlings and juvenile animals were the most affected. In that case, contaminated horse meat fed to the alligators was presumed to be the source of the outbreak. [211]. WNV has also been associated with lymphohistiocytic proliferative cutaneous lesions in American alligators. Round to ovoid lesions were described in the superficial dermis of infected alligators. The lesions were composed of large numbers of lymphocytes and macrophages. No virus was detectable in the lesions, but animals with lesions consistently tested positive for antibodies against WNV and 97.5% tested positive for WNV RNA in pooled skin and liver-brain samples [213]. In a transmission study with alligators, viremia developed in all alligators infected by subcutaneous injection, with the time to development and duration of viremia dependent on temperature (32 or 27 °C). Tankmates of infected alligators kept at 32 °C also became infected. Viremia also developed after oral infection and also led to the infection of tankmates. High viral loads were found in tissues of animals that died of infection (2 of 27 infected). High viral titers were also found in tissues of diseased animals. Viral titers in serum of infected animals were sufficiently high to infect mosquitoes (*Culex quinquefasciatus*), and high virus loads were also documented in the cloaca [214]. West Nile virus has been detected in mosquitoes associated with alligator farms and in alligator blood detected in mosquitoes that are vectors of West Nile virus [215].

## 18. Togaviridae

The family *Togaviridae* contains spherical virions with a lipid envelope and a linear, positive sense single-stranded RNA genome 9.7–11.8 kb in size. The family is divided into two genera: *Alphavirus* and *Rubivirus* [216]. The togaviruses detected in reptiles to date have all belonged to the genus *Alphavirus*, which contains viruses that have the ability to replicate in and be transmitted horizontally by mosquitoes. Most alphaviruses can infect a wide range of vertebrates, mostly birds and mammals, but several have also been reported in reptiles. Studies on alphaviruses in reptiles have mostly focused on the possible role of these animals for the transmission of alphaviruses to humans and livestock. This has led to a focus on persistence of alphaviruses in reptiles, particularly viral persistence over winter in temperate regions in the absence of mosquito activity (reviewed in [217]). Detection of alphavirus infection in reptiles has been carried out by detection of virus in blood or detection of antibodies. Eastern equine encephalitis (EEE) virus, Western equine encephalitis (WEE) virus, and antibodies against these viruses have been found in various chelonians, lizards and snakes, including a number of wild-caught reptiles in the USA (see [118] for a review). The detection of repeated cycles of viremia in garter snakes (genus *Thamnophis*) indicates that recurrence of viremia in these animals has a cyclical rhythm and appeared to be independent of temperature [218]. Experimental transmission of WEE virus to garter snakes showed that the animals developed a sufficiently high viremia to infect mosquitos following hibernation [219–221]. A transmission study involving subcutaneous injection of spotted turtles (*Clemmys guttata*) with EEE virus led to the development of viremia and neutralizing antibodies in infected turtles [222]. Subcutaneous infection of Texas tortoises (*Gopherus berlandieri*) with WEE virus led to high titer viremia over an extended period of time. Viremia was longer at lower temperatures than at high environmental temperatures [223]. None of the transmission studies described any signs of clinical disease in infected reptiles.

## 19. Multiple Infections and Factors

The factors involved in the development of viral disease in reptiles are, in many cases, unclear. Both host and viral factors are involved. Transmission studies have been carried out with a number of viruses in specific host systems, as described above. However, as our understanding of reptilian viral infections grows and as methods for the detection of these viruses become both more sensitive and specific, it becomes more and more clear that additional factors can also be involved. It has long been known that environmental factors, particularly temperature, influence the immune system of reptiles. Increasing evidence also suggests that other factors can play an important role in the outcome of viral infections in reptiles. For example, higher disease rates of fibropapillomatosis in sea turtles have been shown to occur particularly in watersheds with a high nitrogen footprint, which was hypothesized to affect the immune system, possibly through changes in nutrition [224].

Concurrent infections with multiple infectious agents, including multiple viruses, have been shown to occur and the interactions and effects of these concurrent infections are not yet understood. Although fibropapillomatosis is believed to be caused by a herpesvirus, other viruses have been demonstrated in fiborpapillomatosis positive sea turtles, leading to hypotheses that these might affect the immune system of infected turtles and possibly the course of disease [119]. Concurrent infections have long been described in conjunction with adenovirus infections in various reptiles, particularly coinfection with parvoviruses [91,97,106,115,117], as well as with other infectious agents, e.g., coinfection with adenoviruses, parvoviruses, and coccidial protozoa in bearded dragons with neurological disease [89], or coinfection with amoebiasis as well as neoplasia or amoebiasis and IBD in snakes [92]. Reovirus infections have also been described in conjunction with infections with other agents, e.g., cryptosporidiosis in rough green snakes (*Opheodrys aestivus*) with clinical wasting [140]. Coinfections with reo- and adenoviruses have been documented [104]. It is interesting to note that two different transmission studies carried out with two different reoviruses in two different species of snakes led to very different results: in one case, respiratory disease resulted following infection [150], while in another, no clear disease was caused, despite successful infection of the animals [152]. In a case involving corn snakes (*Pantherophis guttatus*) in Germany, co-infection with PMV, several different adenoviruses and a reovirus was documented [103]. In tortoises, coinfection with herpesviruses and mycoplasma has been described [225,226], and infections with herpesviruses, mycoplasma and picornaviruses are also observed [189]. These multiple infections increasingly highlight the importance of carrying out multiple tests when diagnosing viral disease in reptiles. Molecular methods, especially PCRs, are increasingly used for the diagnosis of viral infections in these animals. These methods are often more sensitive than other methods such as virus isolation in cell culture and electron microscopy, but they are also more specific. This specificity can become a disadvantage in a system in which many factors, including multiple viruses, can play a role in the clinical progression and prognosis of disease, and awareness of these interactions and a better understanding of them are important both for clinicians and for researchers.
